# Prognostic and Therapeutic Significance of BTN3A Proteins in Tumors

**DOI:** 10.7150/jca.57831

**Published:** 2021-05-27

**Authors:** Sihan Chen, Zhangyun Li, Wenyi Huang, Yanyan Wang, Shaohua Fan

**Affiliations:** 1 School of Life Science, Jiangsu Normal University, Xuzhou, Jiangsu, China.; 2 College of Health Science, Jiangsu Normal University, Xuzhou, Jiangsu, China.; 3 Department of Ultrasonic Medicine, Xuzhou First People's Hospital, Jiangsu, China.

**Keywords:** Butyrophilin 3A, Tumor, Prognosis

## Abstract

The Butyrophilin 3A (BTN3A) family is a type I transmembrane protein belonging to the immunoglobulin (Ig) superfamily. The family contains three members: BTN3A1, BTN3A2 and BTN3A3, which share 95% homology in the extracellular domain. The expression of BTN3A family members is different in different types of tumors, which plays an important role in tumor prognosis. Among them, there are many studies on tumor immunity of BTN3A1, which shows that it is essential for the activation of Vγ9Vδ2 T cells, while BTN3A3 is expected to become a potential therapeutic target for breast cancer. Recent studies have shown that the BTN3A family is closely related to the occurrence and development of tumors. Now the BTN3A family has become one of the research hotspots and is expected to become new tumor prediction and treatment targets.

## 1. Introduction

### 1.1. Origin of the BTN3A family

The Butyrophilin (BTN) family is a type I transmembrane protein belonging to the immunoglobulin (Ig) superfamily 1-3. It has structural homology with members of the B7 family at the extracellular domain level, that is, an Ig-like domain with IgV and IgC domains 1, 3, 4. Butyrophilin (BTN) protein was first isolated from milk fat globule in the milk of human, bovine, goat and other species by Werner W. Franke. Because of its close relationship with yellow grease, it was named Butyrophilin (in Latin, butyro means butter) 5.

The BTN3A family, also known as CD277, is a subfamily of Butyrophilin (BTN) molecules, including BTN3A1, BTN3A2 and BTN3A3 6, 7, which are three isomers produced by two successive duplications of the BTN3 gene 8. These three isotypes are encoded by three different genes found in humans and some non-human primates 9. They are expressed in most human immune cell subsets, including T cells, B cells, monocytes, dendritic cells and natural killer (NK) cells 4, 6, 10, as well as hematopoietic and non-hematopoietic tumor cell lines 11, 12. The specific antibody 20.1 can bind to the extracellular domain of BTN3A molecule and induce the activation of Vγ9Vδ2 T cells, while antibody 103.2 can inhibit this process 13, 14.

### 1.2. The structure of the BTN3A family

Each member of the BTN3A family has an extracellular N-terminal IgV domain and a near-membrane IgC domain connected to a one-way transmembrane domain 13, 15, 16. The extracellular domains of the three BTN3A subtypes are structurally similar with 95% homology 9, and there is only a slight angular difference between the IgV and IgC domains 13, 17. The extracellular domains of BTN3A1 and BTN3A3 have 97% sequence identity 2. There are two possible conformations in its static extracellular domain, namely, “head-tail” conformation and “V-shaped” conformation 18, 19, while “V-shaped” conformation is recognized and observed by more people.

**Figure 1:**
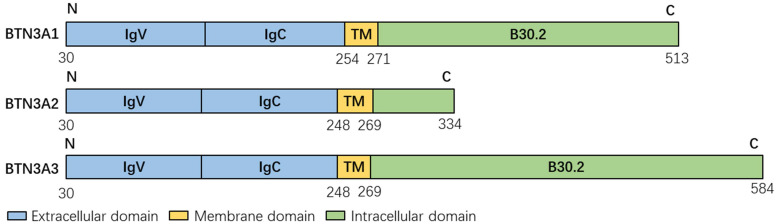
The structure of the members of the BTN3A family. Extracellular domain, blue; Membrane domain, yellow; Intracellular domain, green. The data in this figure is from the Universal Protein Resource (UniProt) website (https://www.uniprot.org/). TM: Transmembrane domain.

The intracellular domains of the three members of the BTN3A family are different (Figure 1). BTN3A1 and BTN3A3 have an intracellular B30.2 domain 6, 11, 20. BTN3A1 has a unique pocket lined with basic amino acid residues, including histidine (His351 and His378), arginine (Arg412, Arg418 and Arg469) and lysine (Lys393), providing a highly positively charged environment that can complement the negative charge of the pyrophosphate portion but position 351 of the B30.2 structure of BTN3A3 is arginine 17, 21. Apart from this, BTN3A3 has a unique C-terminalextension of 70 amino acids 17. However, BTN3A2 lacks the B30.2 domain 6, 17, 22-24. The reason for this phenomenon is that an Alu sequence is translocated into B30.2 exon of BTN3A2 during a stage of primate evolution, resulting in the interruption of reading frames with different intergroup lengths 25.

## 2. BTN3A1 and Cancer

### 2.1. BTN3A1 and prognosis of cancer

The BTN3A family is closely related to many cancers, but its prognostic role is different in different cancers. BTN3A1 is related to the prognosis of ovarian cancer, breast cancer, bladder cancer, pancreatic ductal adenocarcinoma and renal cell carcinoma. BTN3A1 was highly expressed in advanced ovarian cancer 2, but down-regulated in breast cancer 26. The expression of BTN3A1 was positively correlated with the overall survival rate of patients with bladder cancer 27, but negatively correlated with the overall survival rate of patients with pancreatic ductal adenocarcinoma 28. Recently, studies have shown that the response of metastatic clear cell carcinoma to nivorumab can be predicted by the level of BTN3A1 to distinguish between responders who are already at the baseline of treatment and non-responders 29.

### 2.2. BTN3A1 kills tumor cells through the activation of Vγ9Vδ2 T cells

#### 2.2.1. Phosphate antigens are selectively recognized and bound by the intracellular binding domain B30.2 of BTN3A1

Vγ9Vδ2 T cells mainly exist in peripheral blood and can resist microbial infection and cancer 14, 30. Due to the imbalance of mevalonate pathway or the accumulation of intracellular phosphate antigen (pAg) in tumor cells after microbial infection, Vγ9Vδ2 T cells can target tumor cells 31, 32. pAgs include isopentenyl pyrophosphate (IPP), (E)-4-hydroxy-3-methyl-but-2-enyl pyrophosphate (HMBPP) and some synthetic pAgs, such as phosphorylated bromohydrin (BrHPP). The first step in this complex process is the combination of pAg and BTN3A1. Vavassori *et al.* believe that the V-like distal domain of BTN3A1 specifically binds phosphorylated antigens in a shallow groove and then transmits it to Vγ9Vδ2 T cells 14, 18. However, more people think that pAg binds directly to the B30.2 domain of BTN3A1 12, 21, 33-37, but not to the extracellular IgV-like domain. It is worth noting that when treated with 20.1 agonist antibody, BTN3A1, BTN3A2 and BTN3A3 all gave stimulation signals to Vγ9Vδ2 T cells, indicating that their extracellular domains were involved in the activation process 14, 21.

However, only BTN3A1 mediates pAg-induced activation 21, which indicates the importance of the intracellular domain B30.2. Homologues of the B30.2 domain have been found in all primates carrying Vγ9Vδ2 T cells 9. This domain is generally considered to be a protein-protein interaction module, but their binding chaperones are various and there is no obviously conservative binding interface 21. The B30.2 domain in the intracellular domain of BTN3A3 shares 87% homology with BTN3A1 21, but BTN3A3 cannot stimulate Vγ9Vδ2 T cells in a pAg-dependent manner 21, 36. The difference of a pocket residue H351 (His351) between the B30.2 domain of BTN3A1 and BTN3A3 may determine the pAg binding 21. In addition, Gu *et al.* also mentioned that the pocket-side Y352 (Tyr352) in the B30.2 domain is very important for the binding of pAg to the B30.2 domain 19.

There is a positively charged surface bag in the B30.2 domain of BTN3A1, which combines a series of negatively charged small molecules, including pAg 33. Through the nuclear magnetic resonance (NMR) experiment, Salim *et al.* found that BTN3A1 selectively detected pAg, to distinguish pAg from non-antigenic small molecules through the conformational antigen sensor in its B30.2 domain 33. Through the detection of NMR spectra, Gu *et al.* found that the conformation of B30.2 domain in BTN3A1 cells changed after binding to pAg, and most of the residues in or near the pocket of B30.2 domain and a small part of residues located in other regions of B30.2 domain experienced chemical shift perturbation (CSP) 19. The binding of the B30.2 domain of BTN3A1 to pAg can also induce the immobilization of BTN3A1 on the cell surface, which is also a small step in the process of stimulating Vγ9Vδ2 T cells 21. In addition, BTN3A2 and BTN3A3 may enhance the stimulation of BTN3A1 perception by increasing BTN3A1 levels and experiencing the same extracellular domain changes as BTN3A1, but they are not absolutely necessary 38.

#### 2.2.2. Change in the near membrane domain of BTN3A1

The change of BTN3A1 near membrane domain is also an important participant in the process of Vγ9Vδ2 T cell recognition, which strongly affects the activation of Vγ9Vδ2 T cells 39. The change of BTN3A3 near membrane domain can significantly enhance or decrease the reactivity of γδ T cells. This region is identified as a possible dimerization interface and located near the starting position of the B30.2 domain 39, which implies the importance of the transmembrane domain. Some researchers believe that Ser/Thr^296/297^ and Thr^304^ play an important role in the near-membrane domain. The role of Ser/Thr^296/297^ may lie in its ability to stabilize some local folding or its ability to participate in the formation of binding interface with B30.2, while Thr^304^ is involved in binding HMBPP 40. The near membrane domain is flexible in the natural state, but once B30.2 is clamped in combination with HMBPP, it is likely to cause the near membrane domain to become a rigid spiral shape consistent with the compression direction of the whole domain 40. The transmembrane region of BTN3A1 has no specific contribution to the development of Vγ9Vδ2 T cell response 39.

The accumulation of intracellular phosphate antigen leads to the relocation of RhoB from the nucleus or nuclear membrane to the proximal region of the plasma membrane, where it can directly interact with BTN3A1 31. At the same time, RhoB will complete the transition from GDP-bound state to GTP-bound state, that is, from inactive state to active state 31. RhoB can also regulate the membrane mobility of BTN3A1 on cancer cells 31. Recently, it has been confirmed that the decrease of transmembrane protein BTN3A1 migration on target tumor cells is a key determinant of Vγ9Vδ2 T cell activation 31. BTN3A2 regulates the subcellular localization of BTN3A1, and BTN3A2 recombines with BTN3A1 in an IgC-dependent manner 41. If there is endogenous BTN3A1 expression in Vγ9Vδ2 T cells, but lack of BTN3A2 expression, the cells show impaired activation 41, suggesting that BTN3A2 also plays a role in the activation of Vγ9Vδ2 T cells. In addition, the full functional activity of BTN3A1 required BTN3A2, with some capacity of BTN3A3 to substitute for BTN3A2 41.

#### 2.2.3. The extracellular domain of BTN3A1 changed and is recognized by Vγ9Vδ2 T cells

BTN3A1 can form homodimers through its IgC domain 41. BTN3A1 exists on the surface of cancer cells in the form of dimers 39, and can also form heterodimers with BTN3A2 19. RhoB interacts with BTN3A1 homodimer in cancer cells recognized by Vγ9Vδ2 T-cell receptor (TCR) 31. Other studies have shown that the accumulation of phosphate antigen is related to the conformational change of BTN3A1 dimer 31. The increase of intracellular phosphate antigen level can induce the extracellular change of BTN3A1 dimer, which may act as a molecular feature recognized by Vγ9Vδ2 TCR 31. On the other hand, RhoB may dissociate from the fixed BTN3A1 after the binding of phosphorylated antigen to B30.2 and the change of extracellular conformation 31.

**Figure 2:**
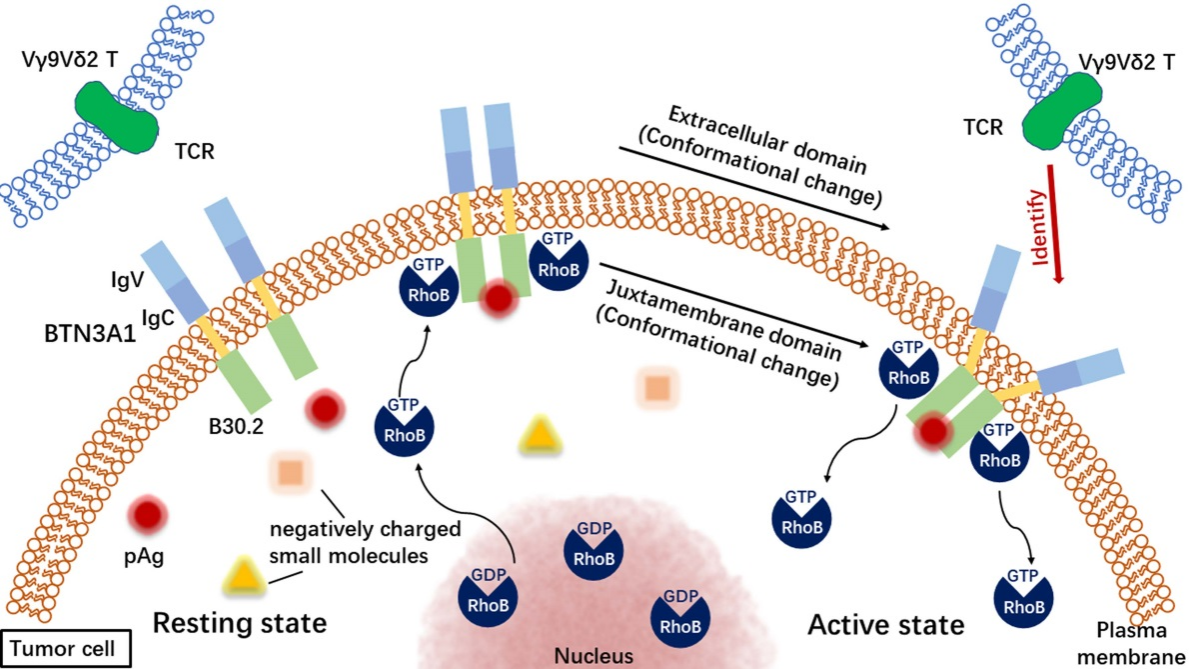
The process of recognizing tumor cells by Vγ9Vδ2 T cells. TCR: T-cell receptor.

In general, pAg binding to BTN3A1 and activating Vγ9Vδ2 T cells is an “inside-out” signal transduction mechanism 39, 42 (Figure 2). Vγ9Vδ2 T cells activated by phosphorylated antigenicity can recognize and kill a variety of tumor cell lines, including breast cancer 43, pancreatic cancer 44, renal cell carcinoma 45-47, melanoma 48, neuroblastoma 49, oral squamous cell carcinoma 50 and multiple myeloma 51, 52. Cancer therapy, which focuses on tumor immune response mediated by γδ T cells, is considered to be a promising treatment 22, 53-55.

### 2.3. Man-made mutation of BTN3A1

At present, no spontaneous mutation of BTN3A family members has been identified or characterized. However, in order to determine the binding region between pAg and BTN3A1 and the importance of some residues, some researchers have constructed mutants of BTN3A1. Salim M *et al*. constructed the mutant of BTN3A1 H381A, H351R, Y382A, H381R and Y382F, and found that compared with wild type (WT) BTN3A1, BTN3A1 H381A, H351R and Y382A reduced the activation of Vδ2^+^ T cells by more than 50% 33. Besides, BTN3A1 H381R mutant reduced the activation of Vδ2^+^ T cells by more than 90%, while the BTN3A1 Y382F could support more than 60% of the activation observed in WT BTN3A1 33. Sandstrom A *et al.* carried out charge-reversal mutations on five basic residues in the positive charge pocket of BTN3A1 B30.2: H378D, K393D, R412E, R418E and R469E 21. It was found that the mutant could not bind to pAg and could not support the stimulation of Vγ9Vδ2 T cells. Gu S *et al.* constructed the BTN3A1 Y352A mutant. Through isothermal titration calorimetric test, it was found that compared with BTN3A1 B30.2, the mutant Y352A had obvious defects in binding to 1-hydroxy-2-methylpent-2-enyl-pyrophosphonate (cHDMAPP) and IPP, resulting in a decrease in T cell activation 19. Nguyen K *et al.* found that the BTN3A1 ST296AA and T304A mutants they constructed had a reduced ability to respond to intracellular ligand treatment to trigger the activation of Vγ9Vδ2 T cells, thus proving the importance of Ser/Thr^296/297^ and Thr^304^ in juxtamembrane region 40. Through the construction of BTN3A1 H351R and BTN3A3 R351H mutants, Sandstrom A *et al.* found that the BTN3A1 H351R mutants could not bind to pAg, but the binding of BTN3A3 R351H and pAg was enhanced, so as to determine the importance of 351 position 21. Gu S *et al.* found that locking BTN3A1 to the V-type dimer interface (D124 / S207C) could significantly prevent pAg-induced and 20.1-induced T cell activation by mutating D124 and S207 residues in the V-type dimer interface to cysteine. This shows that the V-conformation is the resting state of BTN3A1, which is not enough to activate T cells 19.

## 3. BTN3A2 and cancer

The research on BTN3A2 in cancer is mainly focused on the prognosis of cancer, and there is no report on the treatment of cancer. The expression level of BTN3A2 is higher in gastric cancer 56, 57, pancreatic cancer 58 and ovarian cancer 59, but lower in breast cancer 60. The expression of BTN3A2 is related to the adverse progression of many tumors, and its overexpression is related to the increased proliferation and invasion of gastric cancer cell lines, as well as poorly differentiated tumors in patients with pancreatic ductal adenocarcinoma who show short-term survival 58. However, the expression of BTN3A2 is also a marker of good prognosis in many cancer patients. In breast cancer, BTN3A2 is an independent prognostic marker for triple negative breast cancer patients 60. BTN3A2 is positively correlated with immune cell infiltration of CD8+ T cells, T cell (general), DCs, Th1 cells, and T-cell exhaustion 60. BTN3A2 can mediate immune infiltration by regulating T cell receptor interaction and NF-κB signal pathway 60. The higher expression level of BTN3A2 is related to the good prognosis of HR negative, HER2 positive (HR-/HER2+) breast cancer patients, and also to the distant metastasis-free survival (DMFS) rate of breast cancer patients 61. Additionally, the expression of BTN3A2 is related to the prognosis of patients with ovarian cancer 15, 42, overall survival and disease-free progression, and its high expression is closely related to the increased overall survival rate of patients 59.

## 4. BTN3A3 and cancer

### 4.1. BTN3A3 and prognosis of cancer

The single nucleotide polymorphism (SNP) in BTN3A3 was negatively correlated with the risk of ovarian cancer 62, and BTN3A3 was associated with a lower risk of ovarian cancer recurrence 15. Changes in the expression of specific genomes show the response of women with advanced epithelial ovarian cancer to chemotherapy. The expression of BTN3A3 gene can predict early recurrence of ovarian cancer after platinum-paclitaxel chemotherapy (21 months), with an accuracy of 86% 62. However, in breast cancer, some high-grade malignant human breast cancer cell lines (such as MDA-MB-231) express high levels of BTN3A3, while low-grade malignant cell lines (such as MCF-7) express lower levels of BTN3A3 63. BTN3A3 can also predict the sensitivity of patients with gastric cancer to fluorouracil chemotherapy 64. It is reported that BTN3A3 may increase the sensitivity to chemotherapeutic drugs by inhibiting the biological process of EMT 64. BTN3A3 is also associated with colon cancer and can be used as a potential cancer biomarker 65. Compared with healthy controls, the expression of BTN3A3 in patients with ulcerative colitis is significantly up-regulated 66. If ulcerative colitis is not treated as soon as possible, it is likely to develop into colon cancer. In patients with cervical cancer, compared with normal tissues, cervical cancer tissues showed a low level of BTN3A3 methylation, indicating that the immune system of women without cervical cancer may be activated by DNA demethylation 67. According to research, BTN3A3 is associated with intestinal inflammation and colon cancer. There is a negative correlation between the expression of BTN3A3 and IFN-γ in colonic tissue of patients with ulcerative colitis 68.

### 4.2. BTN3A3 and its potential therapeutic strategy for breast cancer

LSECtin is a transmembrane protein highly expressed in tumor-associated macrophages (TAMs), while BTN3A3 is the receptor of LSECtin on breast cancer cells. It has been confirmed that the interaction between LSECtin and BTN3A3 can promote the stem cell characteristics of breast cancer. The extracellular IgC and IgV domains of BTN3A3 are necessary for its interaction with LSECtin, while the intracellular domains of BTN3A3 are necessary for promoting stem cell activity. In mice carrying human tumor xenografts, macrophage-specific LSECtin interference or BTN3A3 interference in breast cancer cells can slow tumor growth. Anti-BTN3A3 mAb destruction of LSECtin-BTN3A3 axis has therapeutic effect on breast cancer. Compared with monotherapy, the combination of anti-BTN3A3 (5E08) mAb and paclitaxel significantly reduced tumor growth. Thus, it can be seen that the LSECtin-BTN3A3 axis may be a unique target in the treatment of breast cancer, which has important clinical significance in the treatment of breast cancer in the future 63.

## 5. Conclusion and prospect

The BTN3A family, also called CD277, consists of three members: BTN3A1, BTN3A2 and BTN3A3. At present, the research on the BTN3A family is mainly focused on the activation of Vγ9Vδ2 T cells. The latest research found that the overexpression of NLR family CARD domain containing 5 (NLRC5) not only increased the levels of BTN3A1, BTN3A2 and BTN3A3, but also promoted the activation and killing of Vγ9Vδ2 T cells functionally. In contrast, CRISPR/Cas9-mediated knockout of BTN3A molecules almost eliminated the enhanced lethality induced by NLRC5 overexpressing cells. This new discovery about the relationship between BTN3A family and NLRC5 further discusses the mechanism of BTN3A family in the activation of Vγ9Vδ2 T cells, and brings more implications for the study of immunotherapy 69.

As the first member of the BTN3A family, the essential role of BTN3A1 in the activation of Vγ9Vδ2 T cells has been verified many times. The latest research shows that CD277 specific antibodies can transform BTN3A1 from immunosuppressive molecules to immunostimulatory molecules, thus dynamically stimulating anti-tumor immunity driven by αβ and γδ T cells to block the progression of established ovarian tumors 53. Therefore, targeting BTN3A1 can exert the synergistic killing effect of αβ and γδ T cells on tumors, and may provide a therapeutic strategy for resisting the existing immunotherapy. Although there are few studies on BTN3A2 and BTN3A3 in tumors, more and more evidence shows that BTN3A2 and BTN3A3 also play an important role in tumors. For BTN3A2, it can mediate immune infiltration by regulating T cell receptor interaction and NF-κB signal pathway 60. For BTN3A3, it can promote the stemness of breast cancer through its interaction with LSECtin 63.

Current studies have shown that members of the BTN3A family are expressed in a variety of tumors, and the expression levels are quite different in different types of tumors. In addition, the primary phenotypic mechanisms of BTN3A family in different tumors are also different (Table 1). This suggests that the BTN3A family has a high complexity in tumorigenesis and development. In a word, BTN3A family is closely related to tumorigenesis and development. With the deepening of its research, BTN3A family is expected to become a new tumor prediction and treatment target, and will have a broad application prospect in the field of tumor.

## Figures and Tables

**Table 1:** BTN3A family and their primary mechanism of phenotype in different tumor types.

Name	Cancer type	*In vitro/in vivo*	Primary mechanism of the phenotype	Reference
**BTN3A1**	Ovarian cancer	*In vitro*	BTN3A1 inhibits the TCR-mediated proliferation of activated human T cells.	2
	Colorectal cancer	*In vitro*	Zoledronate activates colorectal cancer cells in the presence of BTN3A1 to trigger Vδ2 T cells.	22
	Renal cancer	*In vivo*	BTN3A1 can predict response to nivolumab in metastatic clear cell renal carcinoma.	29
**BTN3A2**	Gastric cancer	*In vitro*	BTN3A2 promotes gastric cancer cell proliferation and invasion.	56
	Pancreatic cancer	*In vitro*	BTN3A2 participates in Vγ9Vδ2 T cells anti-tumor functions towards pancreatic ductal adenocarcinoma.	58
	Ovarian cancer	*In vitro, in vivo*	Epithelial expression of BTN3A2 may modulate the infiltration of immune cells with the tumor.	59
**BTN3A3**	Breast cancer	*In vitro*	BTN3A3 enhances the stemness of breast cancer by interacting with LSECtin.	63
	Colon cancer	*In vivo*	The increase of BTN3A3 expression is related to the decrease of IFN-γ level.	68

## Abbreviations

BTN: Butyrophilin
BTN3A: Butyrophilin 3A
BTN3A1: Butyrophilin Subfamily 3 Member A1
BTN3A2: Butyrophilin Subfamily 3 Member A2
BTN3A3: Butyrophilin Subfamily 3 Member A3
Ig: immunoglobulin
NK: natural killer
His: histidine
Arg: arginine
Lys: lysine
Tyr: tyrosine
TM: transmembrane domain
pAg: phosphate antigen
IPP: isopentenyl pyrophosphate
HMBPP: (E)-4-hydroxy-3-methyl-but-2-enyl pyrophosphate
BrHPP: phosphorylated bromohydrin
NMR: nuclear magnetic resonance
CSP: chemical shift perturbation
RhoB: Ras Homolog Family Member B
TCR: T-cell receptor
cHDMAPP: 1-hydroxy-2-methylpent-2-enyl-pyrophosphonate
WT: wild type
HR-: hormone receptor negative
HER2+: human epidermal growth factor receptor 2 positive
DMFS: distant metastasis-free survival
SNP: single nucleotide polymorphism
TAMs: tumor-associated macrophages
NLRC5: NLR family CARD domain containing 5

## References

[1] Cavaletto M, Giuffrida MG, Fortunato D, Gardano L, Dellavalle G, Napolitano L (2002). A proteomic approach to evaluate the butyrophilin gene family expression in human milk fat globule membrane. Proteomics.

[2] Cubillos-Ruiz JR, Martinez D, Scarlett UK, Rutkowski MR, Nesbeth YC, Camposeco-Jacobs AL (2010). CD277 is a negative co-stimulatory molecule universally expressed by ovarian cancer microenvironmental cells. Oncotarget.

[3] Afrache H, Gouret P, Ainouche S, Pontarotti P, Olive D (2012). The butyrophilin (BTN) gene family: from milk fat to the regulation of the immune response. Immunogenetics.

[4] Arnett HA, Viney JL (2014). Immune modulation by butyrophilins. Nat Rev Immunol.

[5] Franke WW, Heid HW, Grund C, Winter S, Freudenstein C, Schmid E (1981). Antibodies to the major insoluble milk fat globule membrane-associated protein: specific location in apical regions of lactating epithelial cells. J Cell Biol.

[6] Messal N, Mamessier E, Sylvain A, Celis-Gutierrez J, Thibult ML, Chetaille B (2011). Differential role for CD277 as a co-regulator of the immune signal in T and NK cells. Eur J Immunol.

[7] Henry J, Ribouchon M, Depetris D, Matteï M, Offer C, Tazi-Ahnini R (1997). Cloning, structural analysis, and mapping of the B30 and B7 multigenic families to the major histocompatibility complex (MHC) and other chromosomal regions. Immunogenetics.

[8] Afrache H, Pontarotti P, Abi-Rached L, Olive D (2017). Evolutionary and polymorphism analyses reveal the central role of BTN3A2 in the concerted evolution of the BTN3 gene family. Immunogenetics.

[9] Harly C, Peigné CM, Scotet E (2015). Molecules and mechanisms implicated in the peculiar antigenic activation process of human Vγ9Vδ2 T cells. Front Immunol.

[10] Yamashiro H, Yoshizaki S, Tadaki T, Egawa K, Seo N (2010). Stimulation of human butyrophilin 3 molecules results in negative regulation of cellular immunity. J Leukocyte Biol.

[11] Compte E, Pontarotti P, Collette Y, Lopez M, Olive D (2004). Frontline: Characterization of BT3 molecules belonging to the B7 family expressed on immune cells. Eur J Immunol.

[12] Rhodes DA, Chen HC, Price AJ, Keeble AH, Davey MS, James LC (2015). Activation of human γδ T cells by cytosolic interactions of BTN3A1 with soluble phosphoantigens and the cytoskeletal adaptor periplakin. J Immunol.

[13] Palakodeti A, Sandstrom A, Sundaresan L, Harly C, Nedellec S, Olive D (2012). The molecular basis for modulation of human Vγ9Vδ2 T cell responses by CD277/Butyrophilin-3 (BTN3A)-specific antibodies. J Biol Chem.

[14] Starick L, Riano F, Karunakaran MM, Kunzmann V, Li J, Kreiss M (2017). Butyrophilin 3A (BTN3A, CD277)-specific antibody 20.1 differentially activates Vγ9Vδ2 TCR clonotypes and interferes with phosphoantigen activation. Eur J Immunol.

[15] Blazquez JL, Benyamine A, Pasero C, Olive D (2018). New insights into the regulation of γδ T cells by BTN3A and other BTN/BTNL in tumor immunity. Front Immunol.

[16] Rhodes DA, Stammers M, Malcherek G, Beck S, Trowsdale J (2001). The cluster of *BTN* genes in the extended major histocompatibility complex. Genomics.

[17] Adams EJ, Gu S, Luoma AM (2015). Human gamma delta T cells: evolution and ligand recognition. Cell Immunol.

[18] Vavassori S, Kumar A, Wan GS, Ramanjaneyulu GS, Cavallari M, El Daker S (2013). Butyrophilin 3A1 binds phosphorylated antigens and stimulates human γδ T cells. Nat Immunol.

[19] Gu S, Sachleben JR, Boughter CT, Nawrocka WI, Borowska MT, Tarrasch JT (2017). Phosphoantigen-induced conformational change of butyrophilin 3A1 (BTN3A1) and its implication on Vγ9Vδ2 T cell activation. Proc Natl Acad Sci U S A.

[20] Nerdal PT, Peters C, Oberg HH, Zlatev H, Lettau M, Quabius ES (2016). Butyrophilin 3A/CD277-dependent activation of human γδ T cells: accessory cell capacity of distinct leukocyte populations. J Immunol.

[21] Sandstrom A, Peigné CM, Léger A, Crooks JE, Konczak F, Gesnel MC (2014). The intracellular B30.2 domain of butyrophilin 3A1 binds phosphoantigens to mediate activation of human Vγ9Vδ2 T cells. Immunity.

[22] Zocchi MR, Costa D, Venè R, Tosetti F, Ferrari N, Minghelli S (2017). Zoledronate can induce colorectal cancer microenvironment expressing BTN3A1 to stimulate effector γδ T cells with anti-tumor activity. Oncoimmunology.

[23] Poggi A, Zocchi MR (2014). γδ T lymphocytes as a first line of immune defense: old and new ways of antigen recognition and implications for cancer immunotherapy. Front Immunol.

[24] Benyamine A, Le Roy A, Mamessier E, Gertner-Dardenne J, Castanier C, Orlanducci F (2016). BTN3A molecules considerably improve Vγ9Vδ2T cells-based immunotherapy in acute myeloid leukemia. Oncoimmunology.

[25] Tazi-Ahnini R, Henry J, Offer C, Bouissou-Bouchouata C, Mather IH, Pontarotti P (1997). Cloning, localization, and structure of new members of the butyrophilin gene family in the juxta-telomeric region of the major histocompatibility complex. Immunogenetics.

[26] Fang J, Chen F, Liu D, Gu F, Chen Z, Wang Y (2020). Prognostic value of immune checkpoint molecules in breast cancer. Bioscience Rep.

[27] Dobosz P, Stempor PA, Roszik J, Herman A, Layani A, Berger R (2020). Checkpoint genes at the cancer side of the immunological synapse in bladder cancer. Transl Oncol.

[28] Bian B, Fanale D, Dusetti N, Roque J, Pastor S, Chretien AS (2019). Prognostic significance of circulating PD-1, PD-L1, pan-BTN3As, BTN3A1 and BTLA in patients with pancreatic adenocarcinoma. Oncoimmunology.

[29] Incorvaia L, Fanale D, Badalamenti G, Porta C, Olive D, Luca ID (2020). Baseline plasma levels of soluble PD-1, PD-L1, and BTN3A1 predict response to nivolumab treatment in patients with metastatic renal cell carcinoma: a step toward a biomarker for therapeutic decisions. Oncoimmunology.

[30] Gu S, Borowska MT, Boughter CT, Adams EJ (2018). Butyrophilin3A proteins and Vγ9Vδ2 T cell activation. Semin Cell Dev Biol.

[31] Sebestyen Z, Scheper W, Vyborova A, Gu S, Rychnavska Z, Schiffler M (2016). RhoB mediates phosphoantigen recognition by Vγ9Vδ2 T cell receptor. Cell Rep.

[32] Moulin M, Alguacil J, Gu S, Mehtougui A, Adams EJ, Peyrottes S (2017). Vγ9Vδ2 T cell activation by strongly agonistic nucleotidic phosphoantigens. Cell Mol Life Sci.

[33] Salim M, Knowles TJ, Baker AT, Davey MS, Jeeves M, Sridhar P (2017). BTN3A1 discriminates γδ T cell phosphoantigens from non-antigenic small molecules via a conformational sensor in its B30.2 domain. ACS Chem Biol.

[34] Wang H, Henry O, Distefano MD, Wang YC, Räikkönen J, Mönkkönen J (2013). Butyrophilin 3A1 plays an essential role in prenyl pyrophosphate stimulation of human Vγ2Vδ2 T cells. J Immunol.

[35] Wang H, Morita CT (2015). Sensor function for butyrophilin 3A1 in prenyl pyrophosphate stimulation of human Vγ2Vδ2 T cells. J Immunol.

[36] Harly C, Guillaume Y, Nedellec S, Peigné CM, Mönkkönen H, Mönkkönen J (2012). Key implication of CD277/butyrophilin-3 (BTN3A) in cellular stress sensing by a major human γδ T-cell subset. Blood.

[37] Hsiao CHC, Lin X, Barney RJ, Shippy RR, Li J, Vinogradova O (2014). Synthesis of a phosphoantigen prodrug that potently activates Vγ9Vδ2 T-lymphocytes. Chem Biol.

[38] Wang H, Nada MH, Tanaka Y, Sakuraba S, Morita CT (2019). Critical roles for coiled-coil dimers of butyrophilin 3A1 in the sensing of prenyl pyrophosphates by human Vγ2Vδ2 T cells. J Immunol.

[39] Peigné CM, Léger A, Gesnel MC, Konczak F, Olive D, Bonneville M (2017). The juxtamembrane domain of butyrophilin BTN3A1 controls phosphoantigen-mediated activation of human Vγ9Vδ2 T cells. J Immunol.

[40] Nguyen K, Li J, Puthenveetil R, Lin X, Poe MM, Hsiao CHC (2017). The butyrophilin 3A1 intracellular domain undergoes a conformational change involving the juxtamembrane region. FASEB J.

[41] Vantourout P, Laing A, Woodward MJ, Zlatareva I, Apolonia L, Jones AW (2018). Heteromeric interactions regulate butyrophilin (BTN) and BTN-like molecules governing γδ T cell biology. Proc Natl Acad Sci U S A.

[42] Gu S, Nawrocka W, Adams EJ (2015). Sensing of pyrophosphate metabolites by Vγ9Vδ2 T cells. Front Immunol.

[43] Guo BL, Liu Z, Aldrich WA, Lopez RD (2005). Innate anti-breast cancer immunity of apoptosis-resistant human γδ-T cells. Breast Cancer Res Treat.

[44] Kitayama J, Atomi Y, Nagawa H, Kuroda A, Mutoh T, Minami M (1993). Functional analysis of TCR gamma delta+ T cells in tumour-infiltrating lymphocytes (TIL) of human pancreatic cancer. Clin Exp Immunol.

[45] Viey E, Fromont G, Escudier B, Morel Y, Rocha SD, Chouaib S (2005). Phosphostim-activated γδ T cells kill autologous metastatic renal cell carcinoma. J Immunol.

[46] Viey E, Laplace C, Escudier B (2005). Peripheral γδ T-lymphocytes as an innovative tool in immunotherapy for metastatic renal cell carcinoma. Expert Rev Anticancer Ther.

[47] Kobayashi H, Tanaka Y, Yagi J, Osaka Y, Nakazawa H, Uchiyama T (2007). Safety profile and anti-tumor effects of adoptive immunotherapy using gamma-delta T cells against advanced renal cell carcinoma: a pilot study. Cancer Immunol Immunother.

[48] Kabelitz D, Wesch D, Pitters E, Zöller M (2004). Characterization of tumor reactivity of human Vγ9Vδ2 γδ T cells *in vitro* and in SCID mice *in vivo*. J Immunol.

[49] Otto M, Barfield RC, Martin WJ, Iyengar R, Leung W, Leimig T (2005). Combination immunotherapy with clinical-scale enriched human γδ T cells, hu14.18 antibody, and the immunocytokine Fc-IL7 in disseminated neuroblastoma. Clin Cancer Res.

[50] Domae E, Hirai Y, Ikeo T, Goda S, Tsuji K (2018). Human Vγ9Vδ2 T cells show potent antitumor activity against zoledronate-sensitized OSCC cell lines. J BUON.

[51] Kunzmann V, Bauer E, Feurle J, Weissinger F, Tony HP, Wilhelm M (2000). Stimulation of γδ T cells by aminobisphosphonates and induction of antiplasma cell activity in multiple myeloma. Blood.

[52] Mariani S, Muraro M, Pantaleoni F, Fiore F, Nuschak B, Peola S (2005). Effector γδ T cells and tumor cells as immune targets of zoledronic acid in multiple myeloma. Leukemia.

[53] Payne KK, Mine JA, Biswas S, Chaurio RA, Perales-Puchalt A, Anadon CM (2020). BTN3A1 governs antitumor responses by coordinating αβ and γδ T cells. Science.

[54] Lu H, Dai W, Guo J, Wang D, Wen S, Yang L (2020). High abundance of intratumoral γδ T cells favors a better prognosis in head and neck squamous cell carcinoma: a bioinformatic analysis. Front Immunol.

[55] Hoeres T, Smetak M, Pretscher D, Wilhelm M (2018). Improving the efficiency of Vγ9Vδ2 T-cell immunotherapy in cancer. Front Immunol.

[56] Zhu M, Yan C, Ren C, Huang X, Zhu X, Gu H (2017). Exome array analysis identifies variants in SPOCD1 and BTN3A2 that affect risk for gastric cancer. Gastroenterology.

[57] Ni J, Deng B, Zhu M, Wang Y, Yan C, Wang T (2020). Integration of GWAS and eQTL analysis to identify risk loci and susceptibility genes for gastric cancer. Front Genet.

[58] Benyamine A, Loncle C, Foucher E, Blazquez JL, Castanier C, Chrétien AS (2018). BTN3A is a prognosis marker and a promising target for Vγ9Vδ2 T cells based-immunotherapy in pancreatic ductal adenocarcinoma (PDAC). Oncoimmunology.

[59] Page CL, Marineau A, Bonza PK, Rahimi K, Cyr L, Labouba I (2012). BTN3A2 expression in epithelial ovarian cancer is associated with higher tumor infiltrating T cells and a better prognosis. PloS One.

[60] Cai P, Lu Z, Wu J, Qin X, Wang Z, Zhang Z (2020). BTN3A2 serves as a prognostic marker and favors immune infiltration in triple-negative breast cancer. J Cell Biochem.

[61] Han J, Choi YL, Kim H, Choi JY, Lee SK, Lee JE (2017). MMP11 and CD2 as novel prognostic factors in hormone receptor-negative, HER2-positive breast cancer. Breast Cancer Res Treat.

[62] Peedicayil A, Vierkant RA, Hartmann LC, Fridley BL, Fredericksen ZS, White KL (2010). Risk of ovarian cancer and inherited variants in relapse-associated genes. PloS One.

[63] Liu D, Lu Q, Wang X, Wang J, Lu N, Jiang Z (2019). LSECtin on tumor-associated macrophages enhances breast cancer stemness via interaction with its receptor BTN3A3. Cell Res.

[64] Pan J, Dai Q, Xiang Z, Liu B, Li C (2019). Three biomarkers predict gastric cancer patients' susceptibility to fluorouracil-based chemotherapy. J Cancer.

[65] Kiyamova R, Garifulin O, Gryshkova V, Kostianets O, Shyian M, Gout I (2012). Preliminary study of thyroid and colon cancers-associated antigens and their cognate autoantibodies as potential cancer biomarkers. Biomarkers.

[66] Lebrero-Fernández C, Wenzel UA, Akeus P, Wang Y, Strid H, Simrén M (2016). Altered expression of Butyrophilin (BTN) and BTN-like (BTNL) genes in intestinal inflammation and colon cancer. Immun Inflamm Dis.

[67] Gašperov NM, Farkas SA, Nilsson TK, Grce M (2014). Epigenetic activation of immune genes in cervical cancer. Immunol Lett.

[68] Lebrero-Fernández C, Wenzel UA, Akeus P, Wang Y, Strid H, Simrén M (2016). Altered expression of Butyrophilin (BTN) and BTN-like (BTNL) genes in intestinal inflammation and colon cancer. Immun Inflamm Dis.

[69] Dang AT, Strietz J, Zenobi A, Khameneh HJ, Brandl SM, Lozza L (2021). NLRC5 promotes transcription of BTN3A1-3 genes and Vγ9Vδ2 T cell-mediated killing. iScience.

